# Diagnostic Dilemma: Investigating Respiratory Symptoms in a Middle‐Aged Smoker

**DOI:** 10.1002/ccr3.9564

**Published:** 2024-11-22

**Authors:** Nazanin Zeinali Nezhad, Mitra Samareh Fekri, Amirhossein Shahpar, Mohsen Nakhaie, Mana Khazaeli, Mehrdad Farrokhnia, Faranak Salajegheh

**Affiliations:** ^1^ Physiology Research Center Institute of Neuropharmacology, Kerman University of Medical Sciences Kerman Iran; ^2^ Clinical Research Development Unit, Afzalipour Hospital Kerman University of Medical Sciences Kerman Iran; ^3^ Gastroenterology and Hepatology Research Center Institute of Basic and Clinical Physiology Sciences, Kerman University of Medical Sciences Kerman Iran; ^4^ Infectious and Tropical Research Center Kerman University of Medical Sciences Kerman Iran

**Keywords:** bronchoscopy, geriatric, hemoptysis, infectious disease, tuberculosis

## Abstract

This case report presents a diagnostic challenge encountered in a 65‐year‐old male admitted with fever, dyspnea, chest pain, and hemoptysis, alongside constitutional symptoms including weight loss, night sweats, and fatigue. Despite initial suspicion for pulmonary thromboembolism and empirical antibiotic therapy for pneumonia, subsequent bronchoscopic evaluation revealed acute necrotizing granulomatous bronchitis, strongly indicative of endobronchial tuberculosis (TB). This diagnosis emphasizes the importance of considering TB in patients with chronic respiratory symptoms, particularly in high‐risk populations. Management involves initiating multidrug antitubercular therapy, close monitoring, infection control measures, and patient education. Prompt diagnosis and appropriate management are crucial in optimizing outcomes and reducing disease burden in TB.

**Summary**

- This case highlights the importance of considering tuberculosis in the differential diagnosis of patients presenting with chronic respiratory symptoms, particularly in high‐risk populations.
- Prompt diagnosis and appropriate management with multidrug antitubercular therapy, close monitoring, infection control measures, and patient education are crucial in reducing the disease burden of tuberculosis.

## 1 Introduction

Tuberculosis (TB), which is caused by bacteria of the *Mycobacterium tuberculosis* complex, is one of the oldest diseases known to affect humans and a leading cause of death worldwide [1].

Approximately one‐third of the global population is infected with a lifetime risk of 10% for developing TB disease. In 2017, there were 10.4 million reported cases of TB worldwide, corresponding to an incidence rate of 133 cases per 100,000 individuals. Among these cases, 90% were adults over the age of 15, and 64% were male [2].

Iran is considered a high‐burden country for TB, with a notable incidence rate. The endemicity of TB in this region increases the pretest probability of the disease in patients presenting with compatible symptoms and radiological findings [3]. Pulmonary TB is usually a disease having a gradual onset. Fever is the most common observed constitutional symptom which characteristically develops in the late afternoon. There may be other manifestations in up to 75% of cases of pulmonary TB, such as malaise, weakness, unusual fatigue, headache, night sweats and weight loss. This is usually accompanied by caseous necrosis and concomitant caseous liquefaction and cough and purulent sputum which is often associated with mild hemoptysis [4].

Sputum smear microscopy and culture are commonly used for the diagnosis of pulmonary TB. However, it's essential to recognize the limitations of these tests [5, 6]. Smear microscopy has a sensitivity ranging from 36.9% to 55.6% and specificity of around 99%, while culture has higher sensitivity (approximately 80%–90%) but takes longer for results [7, 8]. Importantly, negative smear and culture results do not exclude the diagnosis of TB, especially in cases of paucibacillary disease or extrapulmonary involvement [8].

## 2 Case Report

A 65‐year‐old male was admitted to Afzalipour hospital in Kerman, complaining of fever, worsening dyspnea, pleuritic chest pain and hemoptysis. He also mentioned weight loss, night sweats and fatigue. The patient had been well until 3 months before admission to this hospital, when hemoptysis and fatigue developed. After 6 weeks, the patient experienced night sweats and fevers with temperatures of up to 38.4°C.

The patient's past medical history revealed a diagnosis of chronic bronchitis, for which he was prescribed a metered dose inhaler containing fluticasone and salmeterol. In his habitual history, the patient disclosed a history of cigarette smoking, amounting to 15 pack‐years. No history of opium consumption or other substance use was reported.

On examination, he was ill but not toxic, the heart rate was 108 bpm, the blood pressure 128/76 mmHg, the respiratory rate of 31 breaths per minute and the corrected axillary temperature of 38.6°C. His oxygen saturation was 95% while the patient was breathing ambient air. The weight was 67.4 kg and the body‐mass index (the weight in kilograms divided by the square of the height in meters) was 21.2.

On the head examination, temporal wasting and signs of cachexia were observed. Oropharyngeal examination revealed no evidence of bleeding, ulceration, scar tissue, or tumor. There were no signs of lymphadenopathy, masses, or crepitation on neck examination. Respiratory examination indicated increased respiratory effort with mild accessory muscle use. No chest deformities or asymmetry were noted, though mild tenderness was observed over the chest wall. The breath sounds were decreased with crackles heard predominantly over the left lung base. Regular rate and rhythm with no murmurs appreciated on heart examination. S1 and S2 sounds clear. Other systemic examinations were found to be normal.

Nucleic acid testing of a nasopharyngeal swab was negative for severe acute respiratory syndrome coronavirus 2 (SARS‐CoV‐2). Acid‐fast bacilli smear and culture of the sputum was negative for mycobacteria. Other laboratory test results are summarized in Table 1.

**TABLE 1:** A summary of laboratory test results.

Variable	Reference range	At admission (Day 1)	Day 3	Day 5	Day 7
White blood cell (per μL)	4000–10,000	9300	8500	11,100	9800
Hemoglobin (g/dL)	12–16	13.8	13	12.1	11.7
Hematocrit (%)	39–52	42.2	39.6	38.7	36.3
Mean corpuscular volume (Fl)	80–100	78.1	71.3	77.9	76.7
Platelet count (per μL)	150,000–450,000	436,000	468,000	495,000	532,000
Prothrombin time (s)	13–15	12.5	—	—	—
Partial thromboplastin time (s)	24–36	28	—	—	—
International normalized ratio	0.8–1.1	1	—	—	—
Blood sugar (mg/dL)	< 140	137	—	196	—
Urea (mg/dL)	15–45	26	27	32	28
Creatinine (mg/dL)	0.8	0.8	0.7	0.8	0.7
Creatine phosphokinase (U/L)	24–195	43	—	—	—
Lactate dehydrogenase (U/L)	< 480	242	—	—	—
Sodium (mmol/L)	135–145	133	135	137	139
Potassium (mmol/L)	3.8–5	4	4.4	4	3.9
Troponin I	—	Negative	—	—	—
D‐dimer (g/L)	< 70	718	—	—	—
C‐reactive protein (mg/l)	< 6	153	—	211	—
Erythrocyte sedimentation rate (mm/h)	< 12	90	—	—	84

**FIGURE 1:**
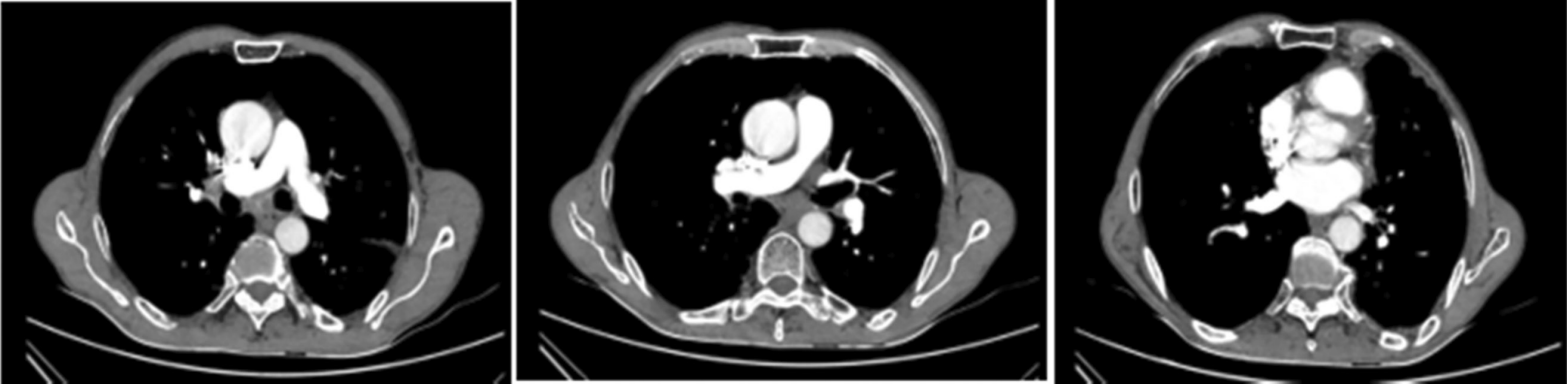
Lung computed tomography angiography (LCTA) revealed no sign of PTE.

Based on the patient's complains (sudden worsening dyspnea and hemoptysis) and wells diagnostic criteria for pulmonary thromboembolism (PTE), the patient's score was 5.5 points (3 points for no alternative diagnosis more likely than PTE, 1.5 points for heart rate > 100 and 1 point for hemoptysis) which revealed an intermediate risk for PTE. The patient underwent lung computed tomography angiography (LCTA) and the result demonstrated that there were no signs of PTE (Figure 1).

**FIGURE 2:**
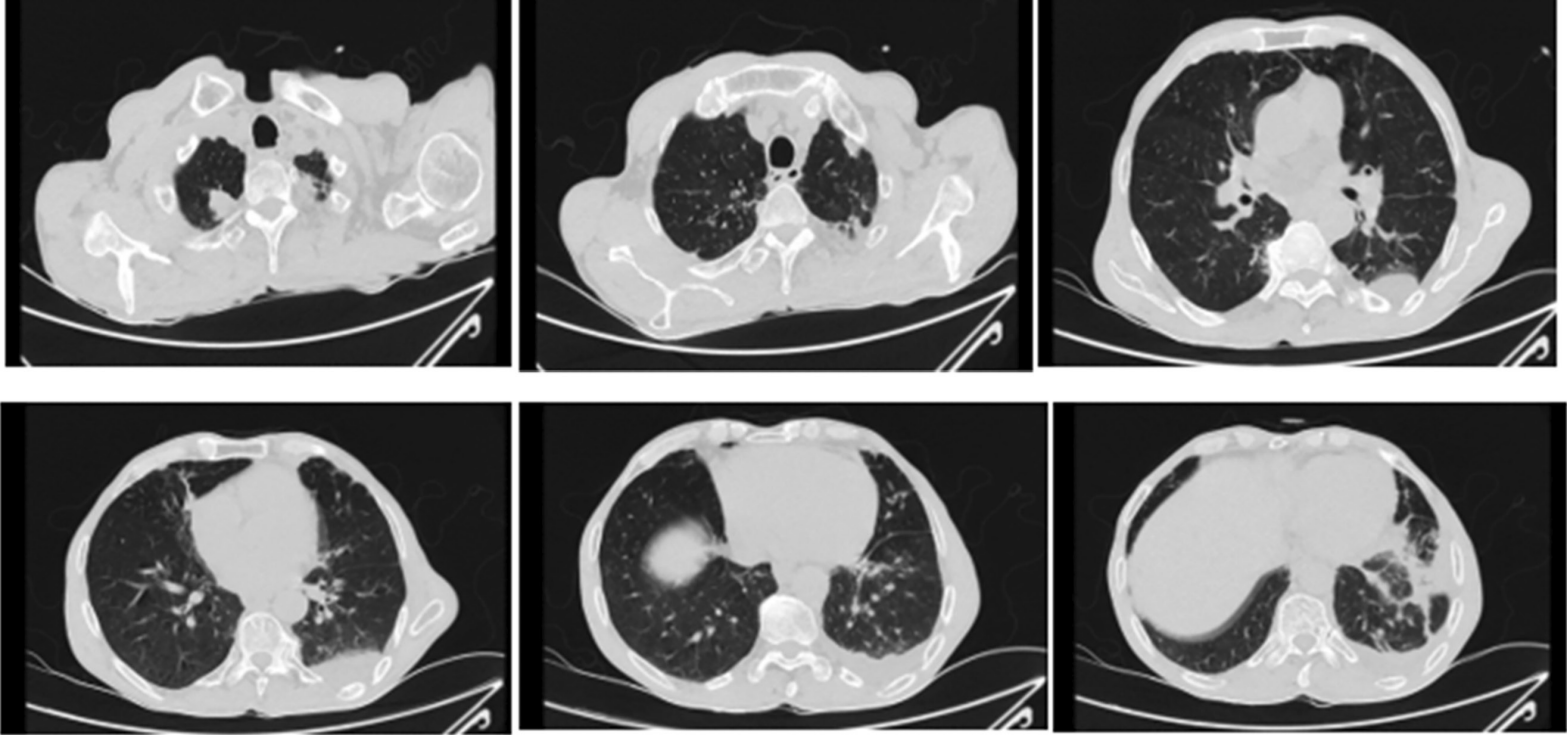
The non‐contrast images from the LCTA. Mild pleural effusion with adjacent lung collapse is seen in LCTA. Collapse consolidation is seen in left lower lobe. Mild pericardiac bronchiectasis is also seen.

After ruling out PTE, the non‐contrast images from the LCTA were carefully reviewed. Based on the patient's risk factors (smoking and age), these images were further evaluated for potential lung pathology. The results demonstrated some bilateral consolidations and bronchiectasis which are shown in Figure 2. So, with the diagnosis of bacterial pneumonia, intravenous normal saline and empirical treatment with ampoule ceftriaxone 1 g every 12 h and ampoule clindamycin 900 mg every 8 h were administered.

After an initial 3‐day period of therapeutic intervention, the patient's fever persisted without significant reduction. Additionally, there was a concerning escalation in the severity of hemoptysis, surpassing the previously observed levels. Consequently, a clinical decision was made to proceed with a bronchoscopy procedure to further investigate and assess the underlying pathology. The bronchoscopy was done on the Day 4 of the patient's hospitalization and the diagnosis was made.

## 3 Differential Diagnosis

This previously healthy 65‐year‐old man presented with fever, dyspnea, pleuritic chest pain, hemoptysis, presence of constitutional symptoms, tachycardia, lung consolidations, bronchiectasis, and elevated levels of inflammatory markers. The elevated levels of inflammatory markers, including the erythrocyte sedimentation rate and levels of C‐reactive protein, are nonspecific findings.

### 3.1 Cancer

Considering the constellation of symptoms presented by the 65‐year‐old male patient, along with radiological findings and relevant history, the probability of lung cancer must be carefully assessed. The patient's chief complaints include hemoptysis, fever, pleuritic chest pain, and weight loss. These symptoms, when observed together, raise significant concern for an underlying malignancy, particularly lung cancer. Hemoptysis, although not pathognomonic for lung cancer, is a concerning symptom often associated with malignancies of the lung parenchyma [9].

The LCTA revealed consolidations in the pulmonary parenchyma. Consolidations, especially when multifocal or persistent, are concerning for neoplastic processes such as lung cancer [10]. While consolidations can be seen in various pulmonary conditions, including infectious etiologies like pneumonia, the presence of consolidations in conjunction with the patient's symptoms and history warrants thorough investigation for malignancy.

Of significant relevance is the patient's history of cigarette smoking. Cigarette smoking is the most significant risk factor for the development of lung cancer, accounting for most cases.

Considering the patient's presentation of hemoptysis, fever, pleuritic chest pain, weight loss, consolidations on imaging, and a history of cigarette smoking, the probability of lung cancer is notably elevated. There are two primary types of lung cancer: non‐small cell lung cancer (NSCLC) and small cell lung cancer (SCLC) [11]. NSCLC is further categorized into three subtypes: adenocarcinoma, squamous cell carcinoma, and large cell carcinoma [12]. Adenocarcinoma is the most common form of lung cancer, particularly among smokers [12]. It tends to occur in the outer regions of the lungs and is associated with a higher incidence in individuals with a history of smoking. Adenocarcinoma often presents with symptoms such as hemoptysis, cough, and weight loss [13].

### 3.2 Infectious Diseases

Considering the clinical presentation, radiological findings, and regional epidemiology, the probability of infectious diseases such as TB and aspergillosis must be carefully considered.

#### 3.2.1 Tuberculosis

Iran is considered a high‐burden country for TB, with a notable incidence rate. The endemicity of TB in this region increases the pretest probability of the disease in patients presenting with compatible symptoms and radiological findings [3]. Sputum smear microscopy and culture are commonly used for the diagnosis of pulmonary TB. However, it's essential to recognize the limitations of these tests [5, 6].

Smear microscopy has a sensitivity ranging from 36.9% to 55.6% and specificity of around 99%, while culture has higher sensitivity (approximately 80%–90%) but takes longer for results [7, 8]. Importantly, negative smear and culture results do not exclude the diagnosis of TB, especially in cases of paucibacillary disease or extrapulmonary involvement [8].

#### 3.2.2 Aspergillosis

Although less common than TB, aspergillosis should also be considered in the differential diagnosis, particularly in immunocompromised individuals. Patients with aspergillosis may present with symptoms like TB, including hemoptysis, fever, and weight loss [14].

TB and aspergillosis should emerge as significant considerations. The high prevalence of TB in Iran, along with the clinical and radiological findings, warrants thorough diagnostic evaluation, including sputum smear microscopy, culture, and potentially imaging‐guided biopsy [15]. It's crucial to recognize the limitations of diagnostic tests and maintain a high index of suspicion, especially in regions endemic for TB.

### 3.3 Pneumonia

Pneumonia typically presents with an acute onset of symptoms, including fever, cough, dyspnea, pleuritic chest pain, and constitutional symptoms like malaise and weight loss [16]. However, in this case, the clinical manifestation began 3 months ago, suggesting a chronic or indolent process rather than an acute infectious etiology. While the presence of consolidations on CT scan is consistent with pneumonia, chronic pneumonia is less common and typically presents with more subtle radiological findings, such as persistent infiltrates or fibrotic changes [16, 17].

Given the chronicity of symptoms and the need to explore alternative diagnoses, further diagnostic evaluation beyond typical pneumonia workup may be warranted. This may include pulmonary function tests, autoimmune serology, imaging studies to assess for structural lung disease or malignancy, and possibly bronchoscopy with biopsy to obtain tissue for histopathological examination.

While pneumonia remains a consideration in the differential diagnosis, its probability is diminished by the chronic nature of the patient's symptoms over the past 3 months.

### 3.4 Interstitial Lung Disease (ILD)

Interstitial lung disease (ILD) encompasses a diverse group of disorders characterized by inflammation and fibrosis of the lung parenchyma, resulting in progressive dyspnea, cough, and impaired gas exchange. Patients may present with constitutional symptoms such as fever and weight loss, particularly in cases of more aggressive or advanced disease. Hemoptysis can occur in ILD, though it is less common compared to other pulmonary conditions [18].

On imaging, ILD typically manifests as reticular opacities, ground‐glass opacities, or honeycombing on CT scan, reflecting interstitial inflammation and fibrosis. Consolidations may also be present, particularly in cases of acute exacerbation or superimposed infection [19].

The presence of chronic symptoms such as hemoptysis, fever, pleuritic chest pain, and weight loss, in conjunction with radiological evidence of consolidations and other interstitial changes, raises suspicion for underlying ILD. The chronicity and progressive nature of the symptoms over time are consistent with the natural history of ILD, which often presents insidiously and worsens gradually [18].

Diagnosis of ILD typically involves a combination of clinical assessment, high‐resolution CT imaging, pulmonary function tests, and sometimes, biopsy for histopathological evaluation. In the context of chronic symptoms such as hemoptysis, fever, pleuritic chest pain, weight loss, and pulmonary consolidations, ILD emerges as a significant consideration [20].

### 3.5 Pulmonary Vasculitis

In the evaluation of the patient, consideration must be given to pulmonary vasculitis, particularly Wegener's granulomatosis too. Wegener's granulomatosis is a systemic autoimmune disorder characterized by necrotizing granulomatous inflammation of the respiratory tract, systemic vasculitis, and glomerulonephritis. Patients may present with constitutional symptoms such as fever, weight loss, and malaise, along with respiratory symptoms including hemoptysis, cough, and pleuritic chest pain. The triad of upper respiratory tract involvement (such as sinusitis), lower respiratory tract involvement (such as hemoptysis and pulmonary infiltrates), and glomerulonephritis constitutes the classic presentation of Wegener's granulomatosis [21].

On imaging, pulmonary vasculitis, including Wegener's granulomatosis, can manifest as multiple nodular opacities, ground‐glass opacities, or consolidations. These findings may be bilateral and involve multiple lung lobes [22].

Diagnosis of Wegener's granulomatosis typically involves a combination of clinical assessment, serological testing (such as anti‐neutrophil cytoplasmic antibodies, or ANCA), imaging studies, and tissue biopsy. ANCA testing, specifically anti‐proteinase 3 (PR3) antibodies, may be positive in up to 80% of cases, aiding in the diagnosis. Tissue biopsy, often obtained via bronchoscopy or surgical lung biopsy, is crucial for confirming the presence of granulomatous inflammation and vasculitis [23].

A comprehensive diagnostic approach, including serological testing and tissue biopsy, is essential for accurate diagnosis and appropriate management of this potentially life‐threatening condition.

## 4 Bronchoscopy Findings

During bronchoscopy examination of the patient, notable findings were observed. Within the trachea and both bronchi, evidence of nodularity was identified. These nodules presented as significant indicators warranting further investigation. The nodules looked quite similar in color to the nearby healthy tissue, making them hard to spot at first. Despite their visual similarity to adjacent healthy mucosa, the nodules' presence raised suspicion for underlying pathology, prompting further investigation.

Considering these findings, a bronchoalveolar lavage (BAL) specimen was collected to assess the cellular composition of the lower respiratory tract. Additionally, multiple biopsies were obtained from the identified nodularities for histopathological examination, aiming to elucidate the underlying pathology driving the patient's clinical presentation. These specimens were processed and sent to the pathology department for detailed analysis. The findings from both the BAL specimen and biopsies are anticipated to provide valuable insights into the nature and extent of pulmonary involvement in this case, guiding subsequent therapeutic interventions and patient management strategies.

## 5 Pathological Findings

**FIGURE 3:**
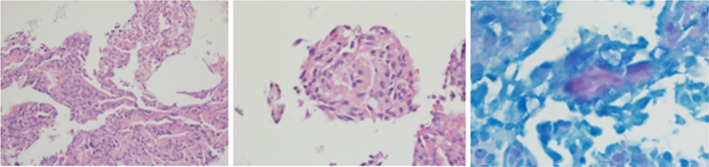
Pathological and Ziehl‐Neelsen staining results.

The examination of the biopsy from the right bronchial mucosa unveiled several significant pathological features. Notably, surface ulceration was observed, indicating tissue damage and disruption. Furthermore, the biopsy exhibited the presence of necrotic tissue, suggestive of tissue death, along with clusters of granulomatous histiocytic cells, indicative of an inflammatory response. Additionally, a few giant cells were noted, further supporting the granulomatous nature of the lesion (Figure 3).

Upon performing Ziehl‐Neelsen staining, focal positivity for acid‐fast bacilli was detected within the specimen, consistent with the presence of *M. tuberculosis* (Figure 3). Notably, the absence of malignant cells or features excluded the possibility of malignancy.

It's important to note that while the initial sputum acid‐fast bacilli smear and culture were negative, TB culture from bronchoscopy samples was performed as part of our standard protocol. This additional step was taken to ensure a comprehensive diagnostic approach, recognizing the limitations of sputum‐based tests alone.

Based on these detailed pathological findings, the diagnosis was established as acute on necrotizing granulomatous bronchitis, strongly indicative of endobronchial and pulmonary TB.

## 6 Discussion of Management

Upon confirming the diagnosis of acute necrotizing granulomatous bronchitis, strongly indicative of endobronchial and pulmonary TB, the patient's management and treatment plan were promptly initiated to address the underlying infectious pathology. The cornerstone of treatment for pulmonary TB involves antitubercular therapy aimed at eradicating the causative agent, *M. tuberculosis* . The patient was commenced on a standard multidrug regimen consisting of first line antitubercular agents, including Isoniazid (INH), Rifampicin (RIF), Pyrazinamide (PZA), and Ethambutol (EMB). The initial phase of treatment typically involves a combination of these drugs administered for a duration of 2 months, followed by a continuation phase with INH and RIF for an additional 4 months [24]. The dosage of each medication was carefully calculated based on the patient's weight and adjusted for any underlying comorbidities or contraindications (Table 2).

**TABLE 2:** First line drugs used in the treatment of adults with TB based on the ATS/CDC/IDSA guidelines.

Anti‐TB drug	Dosage
Isoniazid (INH)	5 mg/kg (~300 mg)
Rifampicin (RIF)	10 mg/kg (~600 mg)
Pyrazinamide (PZA)	1000 mg (40–55 kg weight)
	1500 mg (56–75 kg weight)
	2000 mg (76–90 kg weight)
Ethambutol (EMB)	800 mg (40–55 kg weight)
	1200 mg (56–75 kg weight)
	1600 mg (76–90 kg weight)

Abbreviations: ATS: American Thoracic Society; CDC: Centers for Disease Control; IDSA: Infectious Disease Society of America [25]; TB: tuberculosis.

Given the importance of treatment adherence and the potential for medication noncompliance, a direct observation strategy was implemented to ensure the patient's compliance with the prescribed antitubercular regimen. Healthcare professionals or trained personnel closely monitored and supervised the patient's medication intake to optimize treatment adherence and minimize the risk of treatment failure and drug resistance.

Symptomatic relief measures were incorporated to alleviate the patient's presenting symptoms and improve overall comfort and well‐being. Analgesics such as acetaminophen were administered to alleviate pleuritic chest pain and fever. Hemoptysis management involved bed rest, maintaining airway patency, and monitoring for signs of respiratory distress.

Given the patient's reported weight loss and fatigue, nutritional supplementation and dietary counseling were provided to optimize nutritional status and support immune function during treatment.

Strict infection control measures were implemented to prevent the transmission of *M. tuberculosis* to healthcare workers and other patients. Standard precautions, including airborne precautions, were observed during patient care, particularly during aerosol‐generating procedures such as bronchoscopy.

The patient and their family members were provided with comprehensive education regarding the nature of TB, the importance of treatment adherence, potential adverse effects of medications, and infection control measures to prevent disease transmission.

Close monitoring of the patient's clinical response to treatment, including resolution of symptoms, and laboratory markers of disease activity, was performed at regular intervals. Periodic follow‐up appointments were scheduled to assess treatment adherence, monitor for adverse effects of antitubercular medications.

It is worth acknowledging that our diagnostic approach relied on methods that are widely accessible in our region, such as imaging, bronchoscopy with biopsy, and Ziehl‐Neelsen staining. While advanced diagnostic techniques like metagenomic next‐generation sequencing (mNGS) and TB‐GeneXpert offer superior sensitivity and rapid turnaround time, these technologies are not readily available in many healthcare settings in Iran, including ours, due to resource constraints. This limitation highlights the ongoing challenges in TB diagnosis in resource‐limited settings and underscores the importance of maintaining a high index of clinical suspicion, especially in TB‐endemic regions.

## 7 Conclusion

In summary, the case highlights the diagnostic challenge in patients with pulmonary symptoms. Despite initial suspicion of pulmonary thromboembolism and empirical antibiotic therapy for pneumonia, bronchoscopy revealed findings consistent with acute necrotizing granulomatous bronchitis, indicative of endobronchial and pulmonary TB. Management involves initiating multidrug antitubercular therapy, close monitoring, infection control measures, and patient education. This underscores the importance of considering TB in high‐risk patients and emphasizes the need for prompt diagnosis and appropriate management to optimize outcomes and minimize disease burden.

This case also emphasizes the value of considering TB in patients with atypical presentations, particularly in TB‐endemic regions. It demonstrates how a combination of clinical suspicion and available diagnostic tools can lead to accurate diagnosis even without access to the most advanced technologies. This approach is particularly relevant in resource‐limited settings where advanced diagnostic methods may not be readily available.

## Author Contributions

**Nazanin Zeinali Nezhad:** data curation, methodology, resources, validation. **Mitra Samareh Fekri:** investigation, project administration, visualization, writing – review and editing. **Amirhossein Shahpar:** data curation, methodology, software, writing – original draft. **Mohsen Nakhaie:** formal analysis, visualization, writing – review and editing. **Mana Khazaeli:** data curation, resources. **Mehrdad Farrokhnia:** investigation, project administration, writing – review and editing. **Faranak Salajegheh:** project administration.

## Ethics Statement

All procedures conducted in this study involving human subject adhered to the ethical standards set by both institutional and national research committees, in alignment with the principles outlined in the 1964 Helsinki Declaration and its subsequent amendments, or equivalent ethical standards. This manuscript does not involve any animal studies conducted by any of the authors. Stringent efforts have been made to safeguard patient confidentiality by anonymizing all identifiable information. Patient names, initials, and any other identifying details have been withheld from the report. Moreover, no identifiable images have been included in this publication. Authors encountered no ethical challenges during the case report preparation. The patient's autonomy and privacy were consistently respected, with their medical history and treatment presented with the utmost discretion and sensitivity. The authors reiterate their unwavering commitment to ethical principles in medical publishing, with a primary focus on prioritizing patient welfare, confidentiality, and obtaining written informed consent throughout the entirety of the reporting process.

## Consent

Each author listed assumes responsibility for the overall integrity of the work and has provided consent for the publication of this version. A formal written informed consent has been acquired from the patient, granting permission for the publication of this report in any medical journals.

## Conflicts of Interest

The authors declare no conflicts of interest.

## Data Availability

The data supporting the findings of this case report are available upon request from the corresponding author. Availability of the data is contingent upon journal policy and ethical considerations.
